# Lipid production via simultaneous conversion of glucose and xylose by a novel yeast, *Cystobasidium iriomotense*

**DOI:** 10.1371/journal.pone.0202164

**Published:** 2018-09-12

**Authors:** Ayumi Tanimura, Takashi Sugita, Rikiya Endoh, Moriya Ohkuma, Shigenobu Kishino, Jun Ogawa, Jun Shima, Masako Takashima

**Affiliations:** 1 Division of Applied Life Sciences, Graduate School of Agriculture, Kyoto University, Kyoto, Kyoto, Japan; 2 Department of Microbiology, Meiji Pharmaceutical University, Kiyose, Tokyo, Japan; 3 Japan Collection of Microorganisms, RIKEN BioResource Research Center, Tsukuba, Ibaraki, Japan; 4 Faculty of Agriculture, Ryukoku University, Otsu, Shiga, Japan; Universite Paris-Sud, FRANCE

## Abstract

The yeast strains IPM32-16, ISM28-8s^T^, and IPM46-17, isolated from plant and soil samples from Iriomote Island, Japan, were explored in terms of lipid production during growth in a mixture of glucose and xylose. Phylogenetically, the strains were most closely related to *Cystobasidium slooffiae*, based on the sequences of the ITS regions and the D1/D2 domain of the LSU rRNA gene. The strains were oleaginous, accumulating lipids to levels > 20% dry cell weight. Moreover, kinetic analysis of the sugar-to-lipid conversion of a 1:1 glucose/xylose mixture showed that the strains consumed the two sugars simultaneously. IPM46-17 attained the highest lipid content (33%), mostly C16 and C18 fatty acids. Thus, the yeasts efficiently converted lignocellulosic sugars to lipids, aiding in biofuel production (which benefits the environment, promotes rural jobs, and strengthens fuel security). The strains constituted a novel species of *Cystobasidium*, for which we propose the name *Cystobasidium iriomotense* (type strain ISM28-8s^T^ = JCM 24594^T^ = CBS 15015^T^).

## Introduction

Recently, chemical and fuel production from lignocellulosic biomass is receiving increasing attention [1, 2]. Hydrolysates of such biomasses contain mixtures of sugars, mainly glucose and xylose in various ratios [3, 4]. Complete conversion of sugars in hydrolysates is necessary for efficient utilization of lignocellulosic biomass. The production of microbial lipids via enzymatic degradation of lignocellulosic biomass is currently a subject of intense interest. However, one major barrier to commercial application is the cost of the enzymes used to degrade cellulose to monosaccharides. The absence of a high-performance microorganism (a strain that can efficiently convert released sugars to lipid) renders practical production difficult.

Some yeasts accumulate lipids to over 20% of the cell dry weight and are thus termed oleaginous yeasts [5]. The carbon-chain lengths of the accumulated fatty acids typically range from 12 to 24, with the major components being palmitic acid (C16:0), stearic acid (C18:0), oleic acid (C18:1), and linoleic acid (C18:2) [6, 7]. The composition is similar to that of plant oils [8]; therefore, these lipids can be used as feedstocks for biofuels and oleochemical products. Lignocellulosic biorefineries can achieve greater reductions in CO_2_ emission than petroleum-based biorefineries. However, such refineries are complex and expensive to build [9]. It is anticipated that recent progress in lignocellulosic biorefinery technology will decrease production costs. High-value-added lipids, such as middle-chain fatty acids for use in health foods, are synthesized by oleaginous yeasts. Currently, such lipids are produced from animals and plants; they are expensive and not economically competitive. If lignocellulosic biomass could be used as feedstock, the cost of such lipids could be reduced, and new industries could develop.

Several groups have attempted to produce lipids by culturing oleaginous yeasts on hydrolysates derived from lignocellulosic biomass [10–13]. However, yeasts use glucose in preference to xylose, the uptake of which commences only after glucose is depleted, a phenomenon termed glucose repression [14, 15]. Such sequential utilization prolongs the conversion period and renders the process uneconomic. Efforts have been made to encourage sugar co-conversion, such that glucose and xylose are simultaneously converted to lipids. Zhao et al. [4] optimized the concentrations of sugars, nitrogen sources, and minerals, and achieved a lipid content of 61% dry weight using a medium containing 48.9 g/L glucose and 24.4 g/L xylose. It was concluded that lipid-accumulating ability was influenced by the concentrations of sugars, yeast extract, and FeSO_4_. Unfortunately, the sugar consumption pattern remained unclear, and rigorous preparation of the recommended medium may not be practical. Anschau et al. [16] compared batch, fed-batch, and continuous cultures. Continuous cultivation in a medium with 20 g/L glucose and 45 g/L xylose yielded a high lipid content of 49% dry weight. Both sugar were consumed simultaneously, but half remained in the broth. Glucose repression remains a significant barrier to efficient sugar conversion; optimization of conversion has not yet been achieved. Identification of oleaginous yeasts capable of simultaneous glucose and xylose conversion is thus critical when seeking to improve lipid production efficiency. Efforts have been made to find such yeast. For example, *Cutaneotrichosporon cutaneum* (formerly *Trichosporon cutaneum*) [3, 17] and *Geotrichum fermentans* (formerly *Trichosporon fermentans*) [18] engage in simultaneous glucose and xylose consumption from detoxified lignocellulosic hydrolysates. In addition, it is well-known that the oleaginous yeast *Lipomyces starkeyi* uses glucose and xylose simultaneously to produce lipids [16, 18].

When surveying Japanese isolates, we discovered yeasts exhibiting high-level oleaginous potential under various conditions [17, 19, 20]. In the present study, we focused on oleaginous yeasts that could utilize glucose and xylose simultaneously and selected three strains, IPM32-16, ISM28-8s^T^, and IPM46-17, for evaluation. *These were phylogenetically close to Cystobasidium slooffiae*, *C*. *fimetarium*, and *C*. *minutum*. To assess sugar assimilation patterns, we performed kinetic analyses of the lipid production using a mixture of glucose and xylose. In addition, based on both sequence analyses and phenotypic characterization, we concluded that our strains belonged to a novel species within the genus *Cystobasidium*, for which we propose the name *Cystobasidium iriomotense f*.*a*. sp. nov. (type strain ISM28-8s^T^ = JCM 24594^T^ = CBS 15015^T^).

## Materials and methods

### Strains and media

The new strains were isolated from plant and soil samples collected on Iriomote Island in the Iriomote Ishigaki National Park, Japan [21] (Table 1). A reference strain, *C*. *slooffiae* JCM 10954^T^, was obtained from the Japan Collection of Microorganisms (JCM) at the RIKEN BioResource Center (http://jcm.brc.riken.jp/en/). *C*. *slooffiae* was an appropriate control, because the strain was (1) phylogenetically most closely related to the isolates; (2) capable of glucose and xylose assimilation [22]; and (3) capable of accumulating lipids [23]. YM agar medium (Difco, Detroit, MI, USA) was used for yeast pre-culture and maintenance. The 1:1 (w/w) glucose/xylose (GX) medium was based on the medium of Gong et al. [18], and contained ammonium sulfate 1 g/L, yeast extract 0.5 g/L, potassium dihydrogen phosphate 1 g/L, magnesium sulfate heptahydrate 1 g/L, glucose 10 g/L, and xylose 10 g/L. We used a 1:1 glucose-to-xylose weight ratio to simplify our analyses.

**Table 1:** Yeast strains used in this study.

Species	Strain	Source	JCM/CBS number	Sequence accession no.
*Cystobasidium iriomotense*	IPM32-16	Dead branch of an unidentified tree^a^	JCM 24574	AB726384
	ISM28-8s^T^	Sandy soil^a^	JCM 24594^T^, CBS 15015^T^	AB726571
	IPM46-17	Bark of an unidentified tree^a^	JCM 24575	AB726474
*Cystobasidium slooffiae*	-	Throat swab	JCM 10954^T^	-

JCM, Japan Collection of Microorganisms; CBS, Centraalbureau voor Schimmelcultures

^a^ Samples were collected in November 2008 on Iriomote Island in the Iriomote Ishigaki National Park, Okinawa Prefecture, Japan.

### Sequencing and phylogenetic analysis

DNA fragments, including the internal transcribed spacer (ITS) regions plus the D1/D2 domain of the LSU rRNA gene, were amplified directly from yeast cells. Cells were suspended in 60-μL amounts of Prepman Ultra Sample Preparation Reagent (Applied Biosystems, Foster City, CA, USA) and template DNA prepared according to the manufacturer’s instructions. The ITS regions, including the 5.8S rRNA gene and the D1/D2 domain of the LSU rRNA gene, were amplified using the primers 5’-AACTTGGTCATTTAGAGGAA-3’ [24] and NL4 [25]. The PCR products were directly sequenced using an ABI Prism BigDye Terminator Cycle Sequencing Ready Reaction kit (Applied Biosystems) and analyzed with an Applied Biosystems sequencer model 3100, according to the manufacturer’s instructions. The sequences including the ITS regions and D1/D2 domain of the LSU rRNA gene are available from the DDBJ/GenBank/EMBL database, as shown in Table 1. Reference sequences used in the phylogenetic study were obtained from the DDBJ/GenBank/EMBL database [26]. Multiple alignment was performed using MEGA7 software [27]. A phylogenetic tree was constructed using the maximum likelihood method of MEGA7. The Tamura-Nei model [28] was used for analyses. Initial trees for the heuristic search were automatically obtained by applying the neighbor-joining and BIONJ algorithms to a matrix of pairwise distances estimated using the maximum composite likelihood (MCL) approach and then selecting the topology with the highest log likelihood value. Bootstrap analysis was performed 500 times [29].

### Taxonomic characteristics

Most of the morphological, physiological, and biochemical characteristics were examined as suggested by Kurtzman et al. [30] Assimilation of nitrogen compounds was investigated on solid media using starved inocula. Sexual reproduction tests were performed on YM and cornmeal agar (individual or paired strains) at room temperature.

### Kinetic analysis

Single loops of 3-day-old yeast colonies were suspended in 100 mL amounts of GX medium in Erlenmeyer flasks and incubated at 28°C, with rotary shaking at 150 rpm, for 10 d. Broth was withdrawn at various times. The levels of intracellular lipids and sugars were determined. All experiments were performed in triplicate.

### Measurement of fatty acids

Total intracellular lipid contents were estimated as total fatty acids. Accumulated lipids were extracted from lyophilized cells using a hydrochloric acid-catalyzed direct methylation method [31]. In brief, after cultivation, the centrifuged cells were lyophilized and weighed, dissolved in toluene and methanol, and directly transmethylated with 8% (v/v) methanolic HCl at 100°C for 1 h. The resultant fatty acid methyl esters were extracted with n-hexane and analyzed using a gas chromatograph (GC-2010 Plus; Shimadzu, Kyoto, Japan) equipped with a flame ionization detector (FID) and an autosampler (AOC20; Shimadzu). A TC-17 capillary column (GL Science, Tokyo, Japan) was used. The elution temperature commenced at 165°C for 2 min and then increased by 5°C/min to 180°C, followed by a hold for 5 min, an increase at 5°C/min to 240°C, and an additional hold for 3 min. Helium at 2.0 mL/min served as the carrier gas, and nitrogen as the make-up gas. The injector temperature was 250°C and the detector temperature was 260°C, with a split ratio of 50:1. Major peaks were identified by their retention times using standards obtained from Sigma-Aldrich (St. Louis, MO, USA). Heptadecanoic acid (C17:0) served as an internal standard for the determination of fatty acid concentrations.

### Sugar measurements

Residual glucose and xylose concentrations were determined using a high-performance liquid chromatograph (Shimadzu) equipped with an Aminex Fermentation Monitoring Column (Bio-Rad Laboratories, Hercules, CA, USA) and Micro-Guard Cation H Refill Cartridges in a Standard Cartridge Holder (Bio-Rad Laboratories). The detector was an RID 10A refractive index detector (Shimadzu). The column was held at 60°C using a CTO 20A column oven (Shimadzu). A sulfuric acid solution (5 mM) served as the mobile phase at a constant flow rate of 0.6 mL/min.

## Results

### Phylogeny and phenotypic characteristics

**Fig 1:**
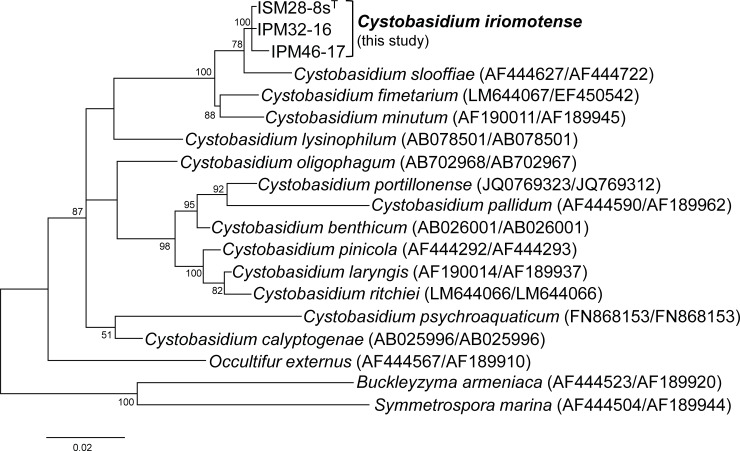
The phylogenetic tree of *Cystobasidium iriomotense* and related species based on the internal transcribed spacer (ITS) regions plus the D1/D2 domain of the LSU rRNA gene. The evolutionary history was inferred using the maximum likelihood method based on the Tamura-Nei model [28]. The tree with the highest log likelihood (-3830.1264) is shown. The rate variation model allowed for some sites to be evolutionarily invariable ([+I], 38.4432% sites). The tree is drawn to scale; the branch lengths indicate the number of substitutions per site. All positions containing gaps and missing data were eliminated. A total of 1,047 positions were present in the final dataset. Bootstrap values < 50% are not shown.

A phylogenetic tree based on the sequences of the ITS regions plus D1/D2 domain of the LSU rRNA genes showed that the three strains clustered with *Cystobasidium slooffiae*, *C*. *fimetarium*, and *C*. *minutum* (Fig 1). The sequence differences in the ITS region ranged from 2 bp (including one gap) to 5 bp (including one gap), and those in the D1/D2 domain ranged from 0 to 2 bp; indicating that the three strains belonged to the same species [32–34]. Of phylogenetically closely related species, the differences between our species and *Cystobasidium fimetarium*, and *C*. *slooffiae* and *C*. *minutum*, were 8–10 bp and 9–11 bp respectively (Fig 1), suggesting that our strains constituted a novel species [33, 35]. In addition, our species was phenotypically distinct from the phylogenetically closely related species *C*. *fimetarium* [36], *C*. *slooffiae* [22, 37] and *C*. *minutum* [22, 37] with respect to several traits: namely, galactose assimilation and the inability to use D-ribose, DL-lactate, or xylitol as the sole carbon source (Table 2). Thus, we propose the name *Cystobasidium iriomotense*. Our strains utilize not only xylose, but also cellobiose or L-arabinose as the sole carbon source (Table 2). We anticipate that they will be industrially useful, but we have not yet determined the lipid accumulation abilities using these sugars.

**Table 2:** Salient characteristics of *Cystobasidium iriomotense* and phylogenetically closely related species.

Species	*C*. *fimetarium*^a^	*C*. *minutum* ^b^	*C*. *slooffiae*^b^	*C*. *iriomotense*		
				IPM32-16	ISM28-8s^T^	IPM46-17
D-Xylose	+	+	+	+	+	+
L-Arabinose	+	+	+	L	+	L
D-Arabinose	+	+	+	LW	LW	LW
Cellobiose	+	+	+	+	+	+
Galactose	-	v	-	L	L	LW
Melezitose	-	+	+	+	+	+
L-Sorbose	-	+	+	LW	LW	LW
D-Ribose	+	+	+	-	-	-
DL-Lactate	+	v	+	-	-	-
Xylitol	+	+	+	-	-	-
Growth at 35°C	-	v	-	LW	-	L
Growth at 37°C	-	-	-	-	-	L

^a^Data from Sampaio and Oberwinkler [36].

^b^*Cystobasidium minutum* and *C*. *slooffiae* were formerly classified as *Rhodotorula minuta* and *R*. *slooffiae*, respectively, and were transferred to the genus *Cystobasidium* by Yurkov et al. [37]. Phenotypic data are from Sampaio [22].

+, positive; -, negative; L, latent; LW, latent and weak; v, variable.

### Lipid accumulation during growth on GX medium

**Fig 2:**
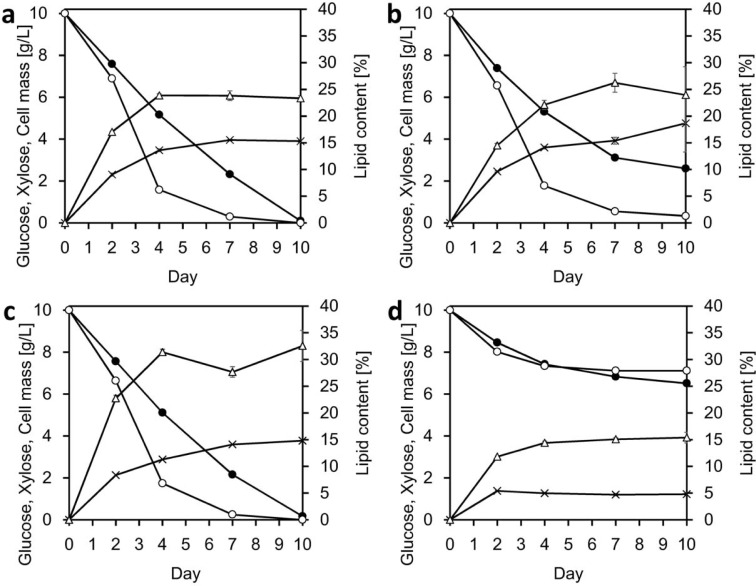
Time course of lipid conversion using glucose/xylose (GX) medium (containing 10 g/L of glucose and 10 g/L of xylose) at 28°C and 150 rpm: glucose (filled circles), xylose (open circles), lipids (open triangles) and cell masses (crosses). (a) IPM32-16; (b) ISM28-8s^T^; (c) IPM46-17; (d) *C*. *slooffiae* JCM 10954^T^. Data are means ± standard deviation (error bars) of three replicates. Some errors are very small and hidden by the symbols.

To explore the time course of conversion of sugars to lipids by IPM32-16, ISM28-8s^T^, IPM46-17, and *C*. *slooffiae* JCM 10954^T^, GX medium containing 10 g/L glucose and 10 g/L xylose as sole carbon sources was used. All strains utilized glucose and xylose simultaneously rather than sequentially (Fig 2). After cultivation, 99.5, 85.3, and 99.2% of the initial sugars were consumed by IPM32-16, ISM28-8s^T^, and IPM46-17, respectively. In contrast, the figure for *C*. *slooffiae* JCM 10954 was 31.9%. In addition, as shown in Fig 2, the curves were biphasic: the log phase persisted from d 0 to 4, followed by the stationary phase from d 4 to 10, except for *C*. *slooffiae* JCM 10954^T^. The strains were similar in terms of lipid content and cell mass (Fig 2). IPM32-16, ISM28-8s^T^, and IPM46-17 yielded lipids to >20% dry cell weight and were thus oleaginous species [38]. *C*. *slooffiae* JCM 10954^T^ yielded lipids to only 15% dry cell weight. Sugar consumption was proportional to lipid production. However, the sugar-converting efficiency of *C*. *slooffiae* JCM 10954^T^ was much lower than those of IPM32-16, ISM28-8s^T^, and IPM46-17. The sugar yields (g lipid per g sugar) of the three strains were 0.046 to 0.064 g/g, whereas that of *C*. *slooffiae* JCM 10954^T^ was 0.03 g/g. Thus, it seems that the strains used different metabolic pathways for lipid production. Nutrients influence lipid production efficiencies because cells use different metabolic pathways depending on the availability of different medium components. A thorough metabolic analysis is required to define the optimal medium for lipid production.

The principal fatty acid in the new strains was oleic acid (C18:1), accounting for 36.3 to 52.8% of all fatty acids in IPM32-16, ISM28-8s^T^, and IPM46-17, whereas the principal fatty acid of *C*. *slooffiae* JCM 10954^T^ was linoleic acid (C18:2) (50.0%) (Table 3, S1 Table for details).

**Table 3:** Fatty acid compositions of IPM32-16, ISM28-8s^T^, IPM46-17 and *C*. *slooffiae* JCM 10954^T^ after 10 d of culture.

Strain	C14:0	C16:0	C16:1	C18:0	C18:1	C18:2	C18:3	C22:0	C24:0
IPM32-16	0.41	20.36	0.12	5.96	52.82	18.25	0.17	0.28	1.64
	8.26	3.49	8.55	3.72	4.56	3.66	72.00	5.72	5.40
ISM28-8s^T^	0.28	17.27	0.03	25.10	44.37	10.55	0.12	0.71	1.57
	4.14	4.56	21.73	2.14	4.02	1.52	8.51	0.28	6.00
IPM46-17	0.77	30.24	0.13	8.13	36.33	22.75	0.17	0.29	1.18
	9.06	6.79	6.86	8.06	6.61	7.05	10.45	9.98	10.60
*C*. *slooffiae* JCM 10954^T^	ND	12.12	0.12	5.67	28.75	50.00	1.05	0.28	2.02
		5.80	6.70	6.25	5.79	2.89	3.49	19.67	8.47

Upper rows: average of three assays [%].

Lower rows: relative standard deviations [%].

ND, not detected.

## Discussion

Simultaneous conversion of glucose and xylose is desirable because xylose is one of the most abundant carbohydrates in plants. Hu et al. [3] investigated the ability of *Trichosporon cutaneum* (present name, *Cutaneotrichosporon cutaneum*) AS 2.571 to produce lipids during cultivation on detoxified corn stover hydrolysate. The strain accumulated lipids to 39.2% of dry cell weight. In another study, *Lipomyces starkeyi* DSM 70296 produced lipids during conversion of a glucose/xylose mixture, to a final content of 27.7% [16]. Huang et al. [39] found that detoxified rice straw hydrolysate could be used for lipid production by *Trichosporon fermentans* (present name, *Geotrichum fermentans*) CICC 1368 (to a lipid content of 23%). When *Trichosporon cutaneum* (present name, *Cutaneotrichosporon cutaneum*) CX1 was cultivated with detoxified corn stover hydrolysate, the final lipid content was 23.5% [40]. The published data on lipid production during growth on a mixture of glucose and xylose are shown in Table 4. We did not observe diauxic growth during cultivation of IPM32-16, ISM28-8s^T^ and IPM46-17 on GX medium. The ability to simultaneously utilize multiple sugars to accumulate lipids is of great importance when planning lipid production from lignocellulosic hydrolysates.

**Table 4:** Conversion of sugar mixtures to lipids.

*Strain*	Substrate	Mode	Initial glucose concentration [g/L]	Initial xylose concentration [g/L]	Lipid content [%]	References
*Cutaneotrichosporon cutaneum (syn*. *Trichosporon cutaneum)* AS 2.571	Detoxified corn stover hydrolysate	Flask	36	25	39.2	[3]
*Geotrichum fermentans (syn*. *Trichosporon fermentans)* CICC 1368	Detoxified rice straw hydrolysate	Flask	15.5	84.3	23	[39]
*Lipomyces starkeyi* DSM 70296	Mixture of glucose and xylose	Fed-batch 2L bioreactor	42	18	27.7	[16]
*Cutaneotrichosporon cutaneum* (syn. *Trichosporon cutaneum)* CX1	Detoxified corn stover hydrolysate	Batch 3L fermenter	44.2	3.92	23.5	[41]
*Lipomyces starkeyi* AS 2.1560	Mixture of glucose and xylose	Flask	47	23	54	[18]

The D1/D2 sequences of ISM28-8s^T^ differed from those of *C*. *fimetarium*, *C*. *minutum*, and *C*. *slooffiae* by 8–9 nucleotides (Fig 1), suggesting that our isolates constituted a new species. Based on the sequence of the ITS region, *C*. *slooffiae* is more closely related to *C*. *iriomotense* ISM28-8s^T^ than are *C*. *fimetarium* and *C*. *minutum*. Therefore, *C*. *slooffiae* JCM 10954 was used as a control strain in the conversion tests, given that the strain accumulates lipids [23] and can use xylose [22]. Indeed, *C*. *slooffiae* JCM 10954^T^ also utilized the sugars simultaneously, but exhibited low-level sugar assimilation and poor lipid production. Interestingly, the three strains preferentially utilized xylose, not glucose (Fig 2A–2C). Generally, transport affinity for glucose is two orders of magnitude higher than that for xylose [40]. However, the sugar transporters of *C*. *iriomotense* remain unknown; further work is thus necessary.

**Fig 3:**
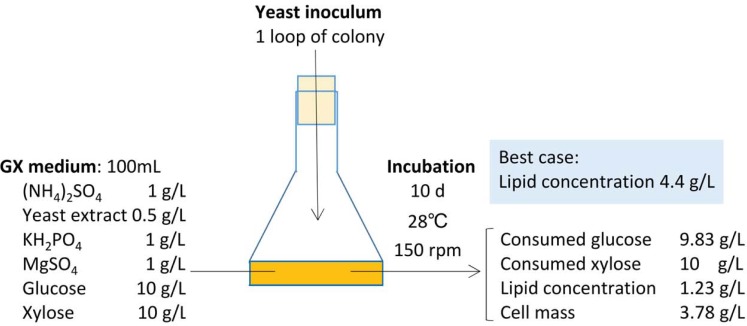
Mass balance of the conversion of glucose and xylose to lipids by *C*. *iriomotense* IPM46-17.

IPM32-16, ISM28-8s^T^ and IPM46-17 accumulated lipids rapidly over the initial 4 d of conversion (Fig 2). For example, the lipid productivity of IPM32-16 (Fig 2A) on d 4 was 0.21 g/L/d, and that over the next 6 d 0.013 g/L/d. After 4 days of conversion, much more xylose than glucose was consumed; the consumption rates were 2.10 g/L/d and 1.21 g/L/d, respectively. From d 4 to d 10, the lipid concentration increased slightly as glucose consumption increased. IPM46-17 behaved similarly, indicating that xylose was efficiently used for lipid production. A similar conclusion was reached in a study on lipid production by *Cutaneotrichosporon curvata* (formerly *Cryptococcus curvatus*, *Candida curvata*) using five different carbon sources: glucose, sucrose, lactose, xylose and ethanol [42]. Fig 3 shows that the final lipid level was 1.23 g/L. In the best practical scenario, the lipid concentration attained 4.4 g/L [9, 43]. Our lipid yield was thus low, probably because we did not consider nitrogen limitation, although nitrogen depletion can induce lipid production. The nitrogen balance and other conditions should be optimized in future.

The fatty acid composition of *C*. *minutum* cultivated on a medium containing 10 g/L glucose was 62% oleic acid (C18:1), 18% palmitic acid (C16:0), 16% linoleic acid (C18:2), and 4% stearic acid (C18:0) [44]. The lipids contained large amounts of C16 and C18 fatty acids (97.4–97.8% of the totals); the lipid mixtures produced were suitable for biodiesel production [45]. Notably, the fatty acid compositions differed among the strains tested. In terms of stearic acid, the lowest level was 5.7% for *C*. *slooffiae* JCM 10954^T^, but ISM28-8s^T^ had a stearic acid content of 25.1%.

We introduce a new yeast species facilitating efficient lipid production; the strain exhibits a unique metabolic profile. Our species may allow for engineering of xylose metabolism in other oleaginous microorganisms. Further analysis and metabolic characterization may aid in the construction of strains efficiently producing lipids from lignocellulosic biomass. Lipid production experiments using a lignocellulosic hydrolysate, such as rice straw, are required.

### Nomenclature

The electronic version of this article in Portable Document Format (PDF), contained in a work with an ISSN or ISBN will represent a published work according to the International Code of Nomenclature for algae, fungi, and plants. Hence, the new names contained in the electronic version of a *PLOS ONE* article are effectively published under that Code from the electronic edition alone; there is no longer any need to provide printed copies.

In addition, new name contained in this work has been submitted to MycoBank, which will make it available to the Global Names Index. The unique MycoBank number and the associated information can be viewed using any standard Web browser by appending the MycoBank number contained in this publication to the prefix http://www.mycobank.org/MB/. The online version of this work is archived and available from the following digital repositories: PubMed Central, LOCKSS.

### Description of *Cystobasidium iriomotense* Tanimura, Sugita et Takashima *f*.*a*. sp. nov.

Etymology: the name *iriomotense* (iri.omot.en’se N.L. adj. iriomote pertaining to Iriomote) was derived from “Iriomote Island”, because the type strain of the species was isolated from soil collected on Iriomote Island.

After 3 days at 25°C in YM broth, the cells are subglobose, oval, ellipsoidal (2.5–5) × (5–10) μm, single, or in pairs. A sediment is formed. After 1 month at 17°C, an incomplete and fragile ring and a sediment are produced. After 1 month at 17°C on YM agar, the streak culture is pastel red, smooth, semi-shiny, and soft to butyrous, with fluid near the bottom, and has an entire margin. Neither mycelium nor pseudomycelium form on YM or cornmeal agar. After 4 weeks of incubation on cornmeal agar at room temperature, ballistoconidia are not produced. Sexual reproduction is not observed on YM or cornmeal agar. Does not ferment glucose. Assimilates glucose, galactose (or latent), L-sorbose (latent and weak), sucrose, cellobiose, trehalose, lactose (or latent), melezitose, ethanol (latent), D-xylose, L-arabinose (or latent), D-arabinose (latent and weak), glycerol, ribitol (latent or latent and weak), D-mannitol (latent), D-glucitol (latent), glucono-δ-lactone (or latent), salicin (variable), succinic acid (latent), D-gluconate, D-glucurono-δ-lactone (latent), propane 1,2 diol, N-acetyl-D-glucosamine, saccharic acid (latent), and xylo-oligosaccharide. Does not assimilate maltose, melibiose, raffinose, inulin, soluble starch, D-ribose, L-rhamnose, erythritol, galactitol, methyl-α-D-glucoside, DL-lactic acid, citric acid, inositol, D-glucuronic acid, D-galacturonic acid, methanol, D-glucosamine, L-arabinitol, quinic acid, xylitol, or butane 2, 3 diol. Assimilates lysine hydrochloride. Does not assimilate sodium nitrite, potassium nitrate, ethylamine hydrochloride, or cadaverine dihydrochloride. Requires *p-* aminobenzoic acid and thiamine for growth. Simultaneously converts glucose and xylose to lipid simultaneously. Grows at 30°C, with variable growth at 35°C and 37°C, but does not grow at 40°C. Starch-like substances are not produced. The diazonium blue B reaction is positive.

**Type strain:** ISM28-8s^T^ (= JCM 24594^T^ = CBS 15015^T^), isolated from soil collected in November 2008 at Iriomote Island in the Iriomote Ishigaki National Park, Okinawa, Japan, by T. Sugita. A culture from the holotype strain of this species has been deposited and preserved in a metabolically inactive state in the Japan Collection of Microorganisms (JCM), RIKEN BioResource Research Center, Tsukuba, Ibaraki Prefecture, Japan; and the Centraalbureau voor Schimmelcultures (CBS), Westerdijk Fungal Biodiversity Institute, Utrecht, the Netherlands.

The Mycobank Number is MB 819779.

## Supporting information

S1 Table GC data. GC data of three separate experiments with the relative standard deviation.
(PDF)
