# Supplementary material for: Lipid production via simultaneous conversion of glucose and xylose by a novel yeast, Cystobasidium iriomotense
Source: PLoS One. 2018 Sep 12;13(9):e0202164. doi: 10.1371/journal.pone.0202164 (PMC6135397; doi:10.1371/journal.pone.0202164)
Supplement: S1 Table — GC data of three separate experiments with the relative standard deviation. (PDF) [file pone.0202164.s001.pdf]

S1 Table. GC data

		C14:0	C16:0	C16:1	C18:0	C18:1	C18:2	C18:3	C22:0	C24:0
IPM32-16	1	0.00402	0.19168	0.00111	0.05540	0.50156	0.16560	0.00282	0.00262	0.01464
	2	0.00385	0.18545	0.00110	0.05189	0.48266	0.16028	0.00088	0.00235	0.01424
	3	0.00341	0.17874	0.00095	0.05536	0.45782	0.17243	0.00092	0.00257	0.01579
	Average of three assays [g/L]	0.00376	0.18529	0.00105	0.05421	0.48068	0.16610	0.00154	0.00251	0.01489
	Relative standard deviations [%]	8.26	3.49	8.55	3.72	4.56	3.66	72.00	5.72	5.40
ISM28-8s	1	0.00304	0.18326	0.00025	0.27044	0.47685	0.11349	0.00125	0.00774	0.01671
	2	0.00288	0.18366	0.00033	0.27028	0.46905	0.11697	0.00115	0.00778	0.01637
	3	0.00312	0.19833	0.00039	0.28049	0.50597	0.11485	0.00137	0.00777	0.01830
	Average of three assays [g/L]	0.00301	0.18842	0.00032	0.27374	0.48396	0.11510	0.00126	0.00776	0.01712
	Relative standard deviations [%]	4.14	4.56	21.73	2.14	4.02	1.52	8.51	0.28	6.00
IPM46-17	1	0.00911	0.34898	0.00165	0.09298	0.42249	0.25775	0.00187	0.00331	0.01287
	2	0.01046	0.39873	0.00173	0.10867	0.47925	0.29624	0.00230	0.00402	0.01592
	3	0.00887	0.36663	0.00151	0.09772	0.43692	0.28428	0.00202	0.00354	0.01468
	Average of three assays [g/L]	0.00948	0.37145	0.00163	0.09979	0.44622	0.27942	0.00206	0.00362	0.01449
	Relative standard deviations [%]	9.06	6.79	6.86	8.06	6.61	7.05	10.45	9.98	10.60
JCM 10954	1	0	0.02399	0.00021	0.01093	0.05442	0.09571	0.00195	0.00064	0.00402
	2	0	0.02137	0.00022	0.00989	0.05066	0.09078	0.00193	0.00047	0.00342
	3	0	0.02291	0.00024	0.01113	0.05686	0.09519	0.00206	0.00046	0.00391
	Average of three assays [g/L]	0	0.02276	0.00022	0.01065	0.05398	0.09389	0.00198	0.00052	0.00379
	Relative standard deviations [%]	−	5.80	6.70	6.25	5.79	2.89	3.49	19.67	8.47
